# Neuroinflammation as a Common Denominator of Complex Diseases (Cancer, Diabetes Type 2, and Neuropsychiatric Disorders)

**DOI:** 10.3390/ijms22116138

**Published:** 2021-06-07

**Authors:** Serena Asslih, Odeya Damri, Galila Agam

**Affiliations:** Department of Clinical Biochemistry and Pharmacology and Psychiatry Research Unit, Faculty of Health Sciences, Ben-Gurion University of the Negev and Mental Health Center, Beer-Sheva 8461144, Israel; serenaa@post.bgu.ac.il (S.A.); odeyad@post.bgu.ac.il (O.D.)

**Keywords:** neuroinflammation, central nervous system (CNS), cancer, type 2 diabetes (T2DM), neuropsychiatric disorders, mood disorders, glia, cytokines, oxidative stress, blood-brain barrier (BBB)

## Abstract

The term neuroinflammation refers to inflammation of the nervous tissue, in general, and in the central nervous system (CNS), in particular. It is a driver of neurotoxicity, it is detrimental, and implies that glial cell activation happens prior to neuronal degeneration and, possibly, even causes it. The inflammation-like glial responses may be initiated in response to a variety of cues such as infection, traumatic brain injury, toxic metabolites, or autoimmunity. The inflammatory response of activated microglia engages the immune system and initiates tissue repair. Through translational research the role played by neuroinflammation has been acknowledged in different disease entities. Intriguingly, these entities include both those directly related to the CNS (commonly designated neuropsychiatric disorders) and those not directly related to the CNS (e.g., cancer and diabetes type 2). Interestingly, all the above-mentioned entities belong to the same group of “complex disorders”. This review aims to summarize cumulated data supporting the hypothesis that neuroinflammation is a common denominator of a wide variety of complex diseases. We will concentrate on cancer, type 2 diabetes (T2DM), and neuropsychiatric disorders (focusing on mood disorders).

## 1. Introduction

Inflammation is a vital host response to the loss of cellular and tissue homeostasis. Inflammation plays a major role in the pathogenesis of metabolic and behavioral abnormalities during cancer, diabetes type II, and neuropsychiatric disorders.

Neuroinflammation is a term used when there is an inflammation of the nervous tissue, in general, and of the central nervous system (CNS), in particular. Neuroinflammation has been suggested as one of the main drivers of neurotoxic symptoms. It is detrimental, and implies that glial cell activation happens prior to neuronal degeneration and, possibly, causes it.

In neuroinflammation, inflammation-like glial responses that do not reproduce classic characteristics of inflammation in the periphery may be initiated in response to a variety of cues, including infection, traumatic brain injury, toxic metabolites, or autoimmunity [1]. Activation of microglia results in their morphological and phenotypical changes and in the release of inflammatory mediators such as cytokines and chemokines [1]. Molfino et al. [2] further suggest that neuroinflammation is both triggered and perpetuated by the activation of microglia, that the release of inflammatory mediators occurs within hypothalamic areas and that the inflammatory response of activated microglia serves to further engage the immune system and initiate tissue repair.

Complex diseases are diseases caused by a combination of genetic, environmental, and lifestyle factors, most of which have not yet been identified. Thus, these diseases do not obey the Mendelian pattern of inheritance, and genetic factors represent only part of the risk associated with complex disease phenotypes. A vast majority of diseases fall into this category, including Alzheimer’s disease, scleroderma, asthma, Parkinson’s disease, multiple sclerosis, osteoporosis, connective tissue diseases, kidney diseases, autoimmune diseases, and more. Intriguingly, a common denominator of many of these disorders is neuroinflammation. 

## 2. Neuroinflammation in Cancer

Inflammation predisposes the development of cancer and promotes all stages of tumorigenesis. Namely, “pro-tumorigenic inflammation promotes cancer by blocking anti-tumor immunity, shaping the tumor microenvironment (TME), and by exerting direct tumor-promoting signals and functions onto epithelial and cancer cells” [3].

### 2.1. Inflammatory Cytokines in Cancer 

Cancer is repeatedly accompanied by depression and cognitive difficulties, the etiology of which remains largely unknown. It is conceivable that the mere realization of the disease might cause changes in mood or that mood problems arise from the toxic side effects of chemotherapy and radiation. On the other hand, tumors themselves might have biological effects on the function of the CNS. In some rodent models, tumor and non-tumor cells in the TME (e.g., leukocytes, fibroblasts, endothelial cells) secrete inflammatory mediators such as interleukin (IL)-1, tumor necrosis factor (TNF)-α, IL-6, IL-8, IFN-α, IL-10, IL-12, TGF-β, and CXCR4 [4,5]. The latter promote tumor development, and, in some cases, transduced, among other tissues, into the brain, leading to neuroinflammation, which, in turn, influences behavior [6]. For example, as shown in rats [7], tumors by themselves are enough to induce impairments in working memory and CNS inflammation, cause a chronic increase in blood cytokine levels and in the expression of brain cytokines, enhance negative or positive feedback on glucocorticoid production, and as a result, generate depressive-like behavior.

Using a large cohort of newly diagnosed breast cancer patients, Patel and colleagues investigated the presence of cancer-related symptoms prior to onset of treatment and the association between symptoms, including neurocognitive performance, and circulating pro-inflammatory cytokine levels [8]. Tumor-induced memory impairment was found to be accompanied by increased expression of hippocampal TNFα mRNA in the brain (though not upregulation in peripheral plasma TNFα). They report that higher plasma levels of a marker of TNFα production was associated with poorer verbal memory but not with impaired executive functioning or processing speed performance. They also found higher IL-1 receptor antagonist (IL-1ra) but not IL-6 levels. Their results suggest that elevated pro-inflammatory cytokines elicited by the underlying disease may be sufficient to induce impaired memory performance. 

Several studies [9,10,11] showed that cancer patients suffer from a high prevalence of depression, anxiety, and cognitive disorders. The fact that these disorders are common among other populations afflicted with chronic inflammatory disease stimulated discussion of potential shared biological mechanisms of neuroinflammation and depressed mood to precede major changes resulting, later, in diagnosis of cancer [12]. Potentially, two mechanisms may be involved—cytokine-related or glucocorticoid responses-related. The relative contribution of the two has yet to be understood. The increase observed in behavioral despair in the absence of important, measurable disease behaviors, indicates a selective effect of tumors on affective behaviors [13]. Together, increases in the production of hippocampal cytokines and GR gene expression suggest that the hippocampus may be a neural substrate in which the endocrine and the inflammation-related cytokines merge to induce depressive behavior in chronic disease, in general, and in cancer, in particular [7]. According to Molfino et al. [14] inflammation and cytokines affect the CNS, and the interaction between inflammatory mediators and the CNS may occur at the periphery, and may play a role in the activation of host inflammatory response which may lead to cancer development. At the periphery, tumor growth might be sensed by the vagal nerve, perhaps by sensing the release of the pro-inflammatory cytokines. Several studies [11,15,16] emphasized that the majority of the postulated mechanisms of mental comorbidities within the cancer context (and other chronic inflammatory diseases) logically focuses on neuroinflammatory pathways. Peripheral tumors and their microenvironment provide the source of various cytokines and, potentially, use neural and/or humoral signaling pathways similar to peripheral infection to gain access to the brain. In addition, Dantzer et al., and Quan and Banks [16,17] focused on models of acute illness. The canonical acute bacterial infection model, a single, sub-toxic i.p. injection of a lipopolysaccharide (LPS) component of the cell wall of the gram-negative bacteria *E. coli*, causes pro-inflammatory cytokine production in the peritoneal cavity. Then, through both neural and humoral signaling pathways, cytokine production rapidly ensues in the brain (hippocampus, hypothalamus, forebrain) and stimulates the production or activation of other inflammatory effectors (IDO, iNOS, Nf-kB, COX). Three other studies [18,19,20] mentioned that the role of these inflammatory markers in cytokine-induced behavioral changes are consistent with clinical research in depressed patients. In summary, they claim that LPS treatment elicits acute sickness behaviors (e.g., lethargy, social withdrawal, fever, anorexia) akin to “somatic” or “vegetative” symptoms of depression and subsequent affective-like behaviors including impaired learning and memory. Experimental manipulation of cytokines in these models (e.g., pharmacologic blockade, cytokine gene knockout) suggests that brain-production of cytokines is necessary and sufficient for subsequent behavioral changes. A recent study in rats bearing bone cancer [21] reported elevated levels of IL-1β, IL-6, and TNF-α and of their respective receptors in the rats’ periaqueductal gray brain region. Prolonged microglia activation leads to the release of the pro-inflammatory cytokines IL-1β, IL-6 and TNF-α, which initiates a pro-inflammatory cascade and subsequently contributes to neuronal damage and losses [22]. Interestingly, blockade of the receptors alleviated the cancer-induced hyperalgesia. This recent report corroborates the previous above-mentioned studies, but further research is still required to improve basic scientific understanding of how activation of pro-inflammatory cytokine networks by cancer cells may increase cancer-related symptoms, to guide clinical interventions.

### 2.2. Cancer, Cytokines and Stress 

Psychosocial stress is highly prevalent in cancer patients and can increase neuroinflammation. Therefore, stress is considered a likely contributor to neurotoxic symptoms. According to Pyter, Brydon, and Woon [13,23,24], in newly diagnosed cancer patients, acute psychological stress is often elevated. Plausible causes are that patients undergo staging and other medical testing, make treatment decisions, cope with current or anticipated physical symptoms, and grapple with existential concerns, all considered to have a major role in neuroinflammation, a predictor of neurotoxic symptoms, both during and after treatment, which may not only affect performance on neurocognitive tests but may themselves activate pro-inflammatory pathways. Indeed, psychological stress may interact with inflammatory pathways to synergistically increase cognitive changes and other behavioral symptoms. Psychological stress may also have direct effects on the CNS, including decreased neurogenesis and hippocampal volume. The latter could be exacerbated by additional biological insults of cancer and its treatment. 

The growing tumor is sensed by the brain via neural, humoral, and inflammatory input. These signals activate the behavioral and metabolic response to stress by activating microglial cells. In turn, microglial activation triggers and perpetuates neuroinflammation by the release of inflammatory mediators within hypothalamic areas. Experimental data suggest that neuroinflammation may contribute to tumor growth and aggressiveness by modulating the peripheral immune response through autonomic output [2].

In breast cancer survivors, Kesler et al. reported an association between lower left hippocampal volume (measured by MRI), higher levels of circulating TNF-α, and lower levels of IL-6 [25]. Similarly, Jenkins et al. reported higher soluble TNF receptor (sTNFR)- 2 and IL-6 levels associated with decreased gray matter volume in specific regions in eight breast cancer patients who underwent chemotherapy during the study [26].

Based on the above, it is tempting to suggest that some of the variance in human mood disorders is comparable to that in cancer, attributable to the effects of tumors by themselves on emotional states. These potential interactions between stress and physiological reactions to cancer warrant further research. 

### 2.3. Cancer, Mitochondria, and Inflammation 

Mitochondria are responsible for cellular energy production by changing adenosine diphosphate (ADP) into adenosine triphosphate (ATP) through aerobic respiration. This conversion occurs in a series of redox reactions in the electron-transport chain-enzyme compounds, labeled complex I–IV. Changes in any of these complexes lead to changes in energy output. Hence, dysfunction in mitochondria and in their DNA (mtDNA) creates reduced cellular energy, which has been linked with neurologic symptoms [27]. Mitochondria are especially vulnerable to oxidative stress, a disturbed balance between reactive oxygen species (ROS), and antioxidants. In turn, mitochondrial dysfunction can lead to ROS overproduction, inducing a downward spiral in cellular functioning [28]. Cancer treatment, inflammation, and stress can all affect mitochondrial function by increasing ROS levels, thereby destroying mitochondria [29]. For example, radiation therapy was shown to increase oxidative stress markers in breast cancer patients with severe acute skin reactions to radiation. In another example, chemo-radiation seemed to normalize tumor-related changes in mtDNA expression in the liver of a rodent model of head and neck cancer, while causing severe changes in mtDNA expression in the brain [30]. This suggests that cancer treatment specifically affects cellular energy production in the CNS. In addition, Lacourt and Heijnen [31] argued that mitochondrial dysfunction is the mechanism leading to neurotoxic symptoms. Both inflammation and stress hormones, as well as cancer treatment, can promote mitochondrial dysfunction, resulting in reduced cellular energy. It should be noted that during cancer many disruptions occur in the physiological functioning of brain areas controlling energy homeostasis [31]. In particular, increased hypothalamic expression and release of mediators of neural inflammation play a major role in this process [2].

In summary, mitochondrial dysfunction may be a final common outcome of cancer, cancer therapy, inflammation, ROS, and stress that leads to neurotoxic symptoms. Mitochondria-protecting drugs preventing cancer therapy-related toxicities may point to promising avenues for treatment of neuro-toxicities in cancer patients. Establishment of these drugs in clinical settings, in parallel to the early implementation of stress-reduction interventions shortly after diagnosis, should be considered to prevent the long-term neurotoxic symptoms that plague so many cancer survivors.

### 2.4. Chemotherapy and Inflammation

Cancer therapy may trigger an inflammatory response through several pathways, including direct immune changes in the TME, tumor cell death, and damage to healthy tissue. Associations between symptoms and inflammatory markers such as IL-6, TNF-α, and C-reactive protein (CRP) have been observed for every treatment modality, both in cross-sectional and in longitudinal designs [32]. In patients who recently completed chemotherapy (compared with treatment-naïve patients), Smith et al. [33] observed reduced blood mononuclear cell DNA methylation associated with higher plasma concentrations of sTNFR2 and IL-6. In turn, sTNFR2 was associated with fatigue, suggesting a transient effect of chemotherapy on inflammation and subsequent fatigue. In paclitaxel-treated mice, Loman et al. [34] found increased fatigue and decreased cognitive performance in parallel with reduced microglial immunoreactivity, increased circulating chemokine (CXCL1) expression, as well as a transient increase in brain gene expression of pro-inflammatory cytokines (IL-1β, TNF-α, IL-6) and CXCL1 in a brain region-dependent manner. The study implied that the brain-gut-microbiota axis was involved in the neuroinflammation induced by the chemotherapeutic agent.

### 2.5. The HPA Axis in Cancer in Relation with Inflammation

Cortisol is released by the hypothalamic-pituitary-adrenal (HPA) axis in response to psychosocial stress. Preliminary evidence indicates that tumors can affect endocrine function [12]. Studies by Pyter et al. [13,35] showed that corticosterone (the primary glucocorticoid in most animals) levels are generally higher in tumor-bearing rodents than in tumor-free controls, and that the hormone’s responsivity to stress is reduced in these animals. Moreover, they reported that growth rates of tumors can be predicted by the production of cytokines, from the constitutive HPA function and according to depressive behavior. The same group made similar observations in patients, although they might have been caused by a combination of psychosocial stress and the tumor. Bower reported a blunted cortisol response in breast cancer survivors along with enhanced inflammatory response to psychological stress [36]. 

Moderate change in the HPA axis has also been found in a variety of CNS-related disorders caused by tumors [37]. In parallel, tumor-bearing mice developed depressive-like behavior along with higher plasma levels of corticosterone, the stress-related hormone [38]. 

In summary, although the mechanism of the interaction between neuroinflammation, systemic inflammation, and tumor growth has not yet been unraveled in full, the relationship between neuroinflammation and cancer-related neuro-toxicities is well-established. Association between inflammatory markers and neuro-toxicities seems to exist even when inflammation is low. It cannot be ruled out that inflammation is only an important mediator in individuals with a genetic vulnerability for exaggerated inflammatory responses to internal (tumor, stress) and external (cancer therapy) stressors. 

## 3. Neuroinflammation in Type 2 Diabetes Mellitus (T2DM)

This section presents data and interpretation of the effect of T2DM on brain structure and function in relation to neuroinflammation. Brain tissue of T2DM patients exhibiting cognitive impairment contains deposits of amylin [islet amyloid polypeptide (IAPP)], a peptide hormone synthesized and co-secreted with insulin by pancreatic β cells [39]. Amylin deposition occurs following chronic over-secretion of amylin (hyper-amylinemia), common in humans with obesity or pre-diabetes insulin resistance. It is toxic, causes pancreatic islet inflammation and is thought to contribute to the development of T2DM [40,41]. Hyper-amylinemia and its consequent oligomerization mediate an inflammatory response, inducing neurological defects [39]. Using a rat model of overexpression of human amylin in the pancreas (the HIP rat) Luchsinger and Nelson et al. [42,43] observed psychomotor speed disturbances followed by full-blown T2DM (blood glucose > 10 nM) accompanied by a significant drop in cognition and memory. Janciauskiene and Ahren and Westwell-Roper et al. [44,45] reported elevation of the pro-inflammatory cytokines TNF-α and IL-6 and down-regulation of the anti-inflammatory cytokine IL-10 in brains from HIP rats. These data support the notion that “human” hyper-amylinemia promotes accumulation of brain oligomerized amylin which, in turn, might trigger an inflammatory response leading to neurological deficits. Using the same HIP rat, Srodulski et al. [39] further describe significantly reduced exploratory drive and impairment in the rotarod test, implicating that infusion of amylin decreases ambulation and locomotion ability in rats. The decline in long-term memory suggests a direct impact of hyper-amylinemia on hippocampal neurons. The authors also found activated microglia, particularly gathering around the small blood vessels in areas positive for amylin infiltration, implicative of neuroinflammation. The latter corroborate the study by Bahniwal et al [46]. They investigated the effects of elevated glucose concentrations (up to 30.5 mM) on functions of cultured human astrocytes in the presence of inflammatory stimuli. Using primary human astrocytes and U-118 MG astrocytoma cells, they found that high glucose increased mRNA expression of IL-6 and secretion of both IL-6 and IL-8 by astrocytes. High glucose also increased the susceptibility of undifferentiated human SH-SY5Y neuronal cells to injury by hydrogen peroxide.

Hyperglycemia in T2DM could contribute to worsening cognitive impairment [47]. This was seen in a rat model of vascular dementia caused by an impaired supply of blood to the brain, and by chronic neuronal inflammation caused by the activation of brain microglia and astrocytes, which might further contribute to neuronal loss [48,49,50]. 

To conclude, T2DM might involve neuroinflammation. Hence, neuroinflammation inhibitors might be novel drugs for this disease. In the case of T2DM induced by brain injury, the list of such targets may also include antioxidants and neurotrophic factors. Since the studies described above imply that brain amylin accumulation might be a pathological substrate for diabetic patients with cognitive decline, reducing blood amylin levels might be another direction for drug design in T2DM. Additional studies are required to clarify the link between brain amylin pathology and impaired cognition, and to search for drugs that will protect the brain in T2DM patients.

## 4. Neuroinflammation in Mood Disorders 

There is growing comprehension of the role of the immune system, in general, and neuroinflammation, in particular, in the pathophysiology of mood disorders. Intriguingly, a comprehensive review and meta-analysis reported that despite a high level of heterogeneity, both monotherapy and add-on anti-inflammatory treatment result in a beneficial effect on depressive symptoms. Nonsteroidal anti-inflammatory drugs (NSAIDs) had a better antidepressant effect [51]. According to Yang et al. [52] neuroinflammation and mitochondrial dysfunction are among the characteristics of psychiatric disorders. Both can lead to increased oxidative stress by excessive release of harmful ROS and reactive nitrogen species (RNS), which further promote neuronal damage and subsequent inflammation. 

The following sections review findings of high cytokine levels as well as possible causes for neuroinflammation in psychiatric disorders.

### 4.1. High Proinflammatory Cytokine Levels

Cumulative data detailed below support the hypothesis that at least in a sub-population of patients afflicted with unipolar depression, the pathophysiology and neurobiological mechanisms underlying resistance to conventional antidepressants stem from high cytokine levels. It also suggests that specific cytokines and their activators and regulators play a role in depression. Indeed, major depression disorder (MDD) and depressed mood are linked with pro-inflammatory cytokines released during periods of disturbance [18]. As nicely elaborated by Jeon and Kim [53], the cytokine hypothesis of depression suggests that cytokine production is initially activated by stress and sympathetic nerve system activation. In turn, cytokines play an important role by acting via neurotransmitter depletion pathways, neuroendocrine pathways, and neural plasticity pathways. There are multiple interactions between these pathways, suggesting existence of a complex model for pathogenesis of depression.

In mouse models of depressive-like behavior, several groups reported hallmark features of neuroinflammation, i.e., microglia and astrocyte reactive morphology, microglial proliferation, increased levels of proinflammatory cytokines, and upregulation of translocator protein (TSPO, a clinical biomarker widely accepted as a surrogate of neuroinflammation that involves an activated state of brain microglia) [54,55,56]. In humans, positron emission tomography (PET) studies [57,58] enhanced TSPO uptake in several brain regions indicated neuroinflammation in depressed patients. 

IL-1β was found to be present in abnormally high levels in plasma, CSF, and postmortem brain tissue of individuals with mood disorders and its levels correlated positively with the severity of depression [59,60]. Accordingly, mRNA levels of proinflammatory cytokines and other related innate immune system proteins were found to be elevated in peripheral blood cells in mood disorder patients [61,62,63,64,65,66], and their plasma and CSF levels were found to be higher during acute depressive episodes, suggesting the pathogenic function of cytokines [62,67]. A meta-analysis based on 29 studies of serum cytokines [68] indicated increased sIL-2R, IL-6, and TNF-α levels, supporting the notion that elevated cytokine proinflammatory levels contribute to the pathophysiology of depression. 

A recent review [69] raised the question whether a specific inflammatory profile underlies suicide risk. The authors summarized that although most studies showed a link between abnormally higher IL-1β, IL-6, TNF-α, transforming growth factor (TGF)-β1, vascular endothelial growth factor (VEGF), kynurenic acid (KYN), and lower IL-2, IL-4, and interferon (IFN)-γ levels in specific brain regions and suicidal behavior, the contribution of MDD as a mediator of the link between these cytokine abnormalities and suicidal behavior could not be excluded. Thus, obviously, additional studies to clarify if, and which immune pathways underlie suicidal behavior are needed.

As for neuroinflammation, in general, and cytokine levels, in particular, in bipolar disorder (BD), a meta-analysis of 30 studies [70] found significantly elevated plasma concentrations of IL-4, IL-6, IL-10, soluble IL-2 receptor (sIL-2R), sIL-6R, TNF-α, sTNFR1, and IL-1 receptor antagonist; IL-1β and IL-6 tended to show higher values in patients. While concentrations of IL-2, IL-4, sIL-6R, and INF-γ were unrelated to medication status, phasic difference was observed for TNF-α, sTNFR1, sIL-2R, IL-6, and IL-1RA, but not for IL-4 and IL-10. The data point at a “cytokine storm” in BD. Nevertheless, the authors of a recent systematic review of 51 articles that measured inflammatory markers in postmortem BD brain samples attested that an absolute statement cannot be concluded whether neuroinflammation is present in BD since a large number of studies did not evaluate the presence of infiltrating peripheral immune cells in the CNS parenchyma, cytokine levels and microglia activation in the same postmortem brain sample [71]. The authors claim that “Future analyses should rectify these potential sources of heterogeneity and reach a consensus regarding the inflammatory markers in postmortem BD brain”.

### 4.2. Glia Pathology

Astroglia and oligodendroglia are essential in neural metabolic homeostasis to maintain behavior and higher cognitive functions. Astroglia and oligodendroglia produce anti-inflammatory cytokines that regulate harmful inflammation [14,72,73]. Animal and human studies report astroglial pathology in psychiatric disorders like MDD and their models [74]. For example, post-mortem studies of MDD subjects implicated reduced oligodendroglial density in the prefrontal cortex and amygdala. Accordingly, it has been suggested that glial loss may contribute to neuroinflammation in psychiatric disorders by several mechanisms [75]. Indeed, using glia-specific genetically modified mice revealed that glial cells such as oligodendrocytes, astrocytes, and microglia affect neuronal function and are involved in the underlying pathobiology of psychiatric disorders [76]. In a model of chronic stress which, as mentioned above, is assumed to be relevant in studying the role of neuroinflammation associated with depression, [77] opted to study whether physiological conditions such as stress enhance susceptibility to inflammation in the substantia nigra, where dopaminergic neuron death occurs in Parkinson’s disease. In a rat model of induced stress and inflammation they found higher TNF-α, IL-1β, IL-6, and iNOS levels in the substantia nigra. Likewise, microglial activation was significantly increased in the infralimbic, cingulate, and medial orbital cortices, nucleus accumbens, caudate putamen, amygdala, and hippocampus of the mice brain following unpredictable chronic mild stress—a reliable model to study depression-induced neuroinflammation [78].

Multiple studies, including some mentioned above, use animal models of depression induced by LPS, which also induces neuroinflammation. In the nonhuman-primate brain, LPS-induced systemic inflammation produces a robust increase in the level of TSPO (detected by PET), reflecting the state of neuroinflammation changes [67]. For example, doxycycline prevented and reversed LPS-induced changes in immobility time on the forced swimming test (FST), and in brain IL1β [79]. Likewise, minocycline also attenuated LPS-induced behavioral changes and markers of neuroinflammation in mice [80].

### 4.3. Increased Oxidative Stress

At the time of microglial activation, pro-inflammatory cytokines and NO production might increase oxidative stress. Namely, pro-inflammatory cytokines and high NO levels may promote ROS formation which, in turn, accelerates lipid peroxidation, damaging membrane phospholipids and their membrane-bound monoamine neurotransmitter receptors and depleting endogenous antioxidants. The consequence of increased production of pro-inflammatory cytokines via stimulation of NF-κB and enhanced microglial activation caused by the increase in ROS products might be increased prevalence of psychiatric disorders [81]. Indeed, studying MMD patients’ fibroblasts, Scapagnini et al. [82] reported an increase in oxidative stress independent of glutathione levels. Moreover, diseases such as MDD, BD, and schizophrenia might go through increased oxidative stress due to mitochondrial dysfunction. Consistent with the high prevalence of psychiatric disturbances in primary mitochondrial disorders, there are reports [82,83,84] of abnormalities in mitochondrial DNA in these disorders. Alternatively, as data in Ott et al. [82,83] imply, there might be mechanistic links among neuroinflammation, mitochondrial dysfunction, and oxidative stress, meriting further investigation of these intersecting pathogenic pathways in human psychiatric disorders.

### 4.4. BBB Dysfunction

MDD-related clinical and experimental studies indicate indirectly that increased oxidation might contribute to endothelial dysfunction. Moreover, oxidation-mediated endothelial dysfunction might contribute to the pathophysiology of BBB dysfunction in psychiatric disorders [85,86]. In a prolonged learned helplessness depression model in mice, the non-recovered group had, within 4 weeks, higher hippocampal levels of TNFα, IL-17A, and IL-23, increased permeability of the BBB and lower levels of the BBB tight junction protein claudin-5 and the tight junction receptors occludin and zonula occludens protein 1 (ZO1), as compared with mice that recovered and with control mice, [87]. 

As for the BBB in BD, in a recent study [88], bipolar patients and control subjects matched for sex, age, and metabolic status underwent contrast-enhanced dynamic MRI scanning to quantitate their BBB leakage. Nearly 30% of the patients exhibited significantly higher percentages of brain volume with BBB leakage. This subgroup had more severe depression and anxiety and a more chronic course of illness.

### 4.5. The Microbiota-Gut-Brain Axis

The microbiota-gut-brain axis (microbiome) is a dynamic matrix of tissues and organs including the brain, glands, gut, immune cells, and gastrointestinal microbiota that communicate in a complex multidirectional manner to maintain homeostasis. It is, thus, regarded as a modulator of various central processes affecting changes in neuroinflammation, as well as neurotransmission and behavior, including stress adaption and immune response. Therefore, gut microbiome dysbiosis might be detrimental, contributing to the development of a number of CNS disorders such as aberrant anxiety and fear responses, despair and anhedonia via mechanisms not yet unraveled. This triggered largely preclinical animal studies investigating the influence of the microbiome, searching for mechanisms by which the microbiome may affect mental health. Some of the studies demonstrate encouraging results in the treatment of depression (for review see [89]), while studies in clinical cohorts have, mostly, been diagnostic in nature, and warrant further ones with pre- and pro-biotic interventions (for review see [90]). 

### 4.6. Microbiota and the BBB

A variety of neuropsychiatric disorders [anxiety, depression, autism spectrum disorders (ASDs), Parkinson’s disease, Alzheimer’s disease, and schizophrenia] have been related to microbial-induced BBB dysfunction [91,92], although the mechanism by which the microbiota affects BBB is unknown. Mediation by gut-derived neurotransmitters and bacterial metabolites is conceivable. Rodent models pointed at a link between microbiota dysbiosis and increased permeability of the BBB, further accompanied by behavioral changes [93], while a pathogen-free gut microbiota restored BBB functionality [91]. 

To summarize this chapter, the data reviewed support the notion that neuroinflammation is a dominant factor which plays a role in the pathophysiology of psychiatric disorders. The field awaits more multidisciplinary efforts to improve basic scientific understanding of whether antidepressant effects might be achieved using therapies for mood disorders that influence neuroinflammation.

## 5. Conclusions

Although cancer, T2DM, and mood disorders, all belonging to the entity of complex disorders, represent diseases of different symptomatology residing in different tissues, perplexingly, they all exhibit neuroinflammation as a common denominator. This awareness may be translated into practice. It may be suggested that drugs known to alleviate neuroinflammation, such as aspirin or lithium, may be repurposed as add-on treatment in these disorders.

## Data Availability

Not applicable.
